# Genome wide association study identifies novel candidate genes for growth and body conformation traits in goats

**DOI:** 10.1038/s41598-022-14018-y

**Published:** 2022-06-14

**Authors:** Muhammad Moaeen-ud-Din, Raja Danish Muner, Muhammad Sajjad Khan

**Affiliations:** 1 Department of Animal Breeding and Genetics, Faculty of Veterinary and Animal Sciences, PMAS-Arid Agriculture University, Rawalpindi, 46300 Pakistan; 2 Cholistan University of Veterinary and Animal Sciences, Bahawalpur, 63100 Pakistan

**Keywords:** Computational biology and bioinformatics, Genetics, Molecular biology

## Abstract

Pakistan is third largest country in term of goat population with distinct characteristics of breeds and estimated population of 78.2 million. Punjab province has 37% of country’s total population with seven important documented goat breeds namely Beetal, Daira Din Pannah, Nachi, Barbari, Teddi, Pahari and Pothwari. There is paucity of literature on GWAS for economically important traits i.e., body weight and morphometric measurements. Therefore, we performed GWAS using 50 K SNP Chip for growth in term of age adjusted body weight and morphometric measurements in order to identify genomic regions influencing these traits among Punjab goat breeds. Blood samples were collected from 879 unrelated animals of seven goat breeds along with data for body weight and morphometric measurements including body length, body height, pubic bone length, heart girth and chest length. Genomic DNA was extracted and genotyped using 50 K SNP bead chip. Association of genotypic data with the phenotypic data was performed using Plink 1.9 software. Linear mixed model was used for the association study. Genes were annotated from *Capra hircus* genome using assembly ARS1. We have identified a number of highly significant SNPs and respective candidate genes associated with growth and body conformation traits. The functional aspects of these candidate genes suggested their potential role in body growth. Moreover, pleiotropic effects were observed for some SNPs for body weight and conformation traits. The results of current study contributed to a better understanding of genes influencing growth and body conformation traits in goat.

## Introduction

Molecular markers, which reveal polymorphisms at the DNA level are now a days key players in animal genetics. However, the choice of molecular markers according to the purpose is crucial viz. which depends upon various molecular biology techniques and their implications^1^. Single nucleotide polymorphisms (SNPs) are DNA sequence variations that occur when a single nucleotide such as adenine (A), thymine (T), cytosine (C) or guanine (G) in the genome sequence is altered. Two main strategies for SNPs genotyping are traditional and high throughput methods. Moreover, SNPs genotyping technologies have become increasingly important in animal breeding programs. Thus, genomic selection using SNPs is a tool for choosing the best breeding animals. Moreover, high density maps using SNPs can provide valuable genetic tools for genetic variation studies of quantitative traits^2^. Eventually technology has been developed which in turn provides valuable tools for SNP discovery and genotyping in non-model organisms^3^.

The status of goat genetics has entirely changed since 2010 after the sequencing of goat genome^4^and release of Caprine SNP50 chip^5^developed by the International Goat Genome Consortium (IGGC). It has also opened a new window of opportunity for genome wide association studies (GWAS). GWAS is widely used to identify genetic variations and candidate regions that are associated with quantitative traits^6–8^. The developments of Illumina Goat 50 K SNP Bead Chip has also provided the opportunity to explore genomic regions that might be under the influence of selection^9^. Moreover studies have been conducted in goats using SNP arrays i.e. native Italian and Moroccan breeds^10^. SNP arrays have also been used for exploring genomic regions, selection signatures and selection history in other worldwide renowned breeds i.e. Alpine, Boer, Cashmere, and Saanen^11^.

Morphological traits are of importance for breed identification, classification and are also positively correlated with body weight. The variations in these traits might be due to selection of breeders for these important traits and environmental effects in different production systems and geographical regions. Consequently the selection process led to goat breeds specialized for milk, meat, fiber or dual purposes^12^. Body weight is also an important trait for successful farming of small ruminants because of its economic value. Therefore, identification of genomic regions for understanding variations in body weight and other morphological traits is quite valuable for selection purpose^13^.

Pakistan has 78.2 million goats making it third largest goat producing country in the world. Goats play a vital role in uplifting the economy of poor farmers in Pakistan. Milk and meat production obtained from goats in Pakistan is 965 and 748 thousand tonnes respectively^14^. Punjab is the largest province of Pakistan with variety of goat breeds. According to the Livestock Census; Punjab has the highest goat population which is 37% followed by Sindh (23%), Balochistan (22%) and KPK (18%) respectively^15^. Beetal, Daira Din Pannah, Nachi (Bikaneri), Barbari, Teddi, Pahari (Kajli) and Pothwari are the documented goat breeds of the province^16^. Goat rearing is commonly meant for meat production as primary objective while milk is also consumed domestically and hair of goats are used for making rugs by poor families. In Pakistan, only fresh meat is usually sold. Hundred thousands of goats are sacrificed on Eid-ul-Azha where Beetal and Kamori breeds are preferred due to their larger size while Teddi which is one of the smallest goat breed is also common because of its highly prolific nature. Moreover, goat rearing is also encouraged by religious customs in certain areas of the country i.e. in Kafiristan goat sacrifice is a custom to celebrate deaths, therefore large flocks of goat are common in these areas^17^.

Despite of having rich diversity of goat breeds in the country; no GWAS has been carried out in Pakistani goats regarding growth and conformation traits. However, Punjab goat breeds have been recently documented using 50 K SNP chip^18^. Current study was aimed to fulfill the gap by finding significant genomic regions or SNPs and relevant genes influencing body weight and morphometric measurements among goat breeds of Punjab province.

## Results

### Descriptive statistics and quality control

Genotyping of DNA samples was performed using 50 K SNP Bead Chip which coverd 53,347 SNPs distributed across the whole Caprine genome. After quality control filters; 36,861 SNPs were left while 11,181 SNPs were removed due to Hardy Weinberg Equilibrium, 3963 removed due to minor allele threshold and 1342 removed due to missing genotypes. Numbers of SNPs present before and after quality controls with average distance in kb (1 kb = 1000 base pairs) at each chromosome are given in Table 1. A total of 52 individuals were removed after quality control measures thus, 827 individuals were used for final GWAS analysis.

**Table 1:** Average distances between adjacent SNPs on each chromosome and distributions of SNPs before and after quality control (QC) measures.

Chromosome	No. of SNPs		Chromosomal length (bp)^a^	Average distance (kb)	
	Before QC	After QC		Before QC	After QC
1	3256	2156	157,403,528	48.34	73.00
2	2829	2122	136,510,947	48.25	64.33
3	2380	1686	120,038,259	50.44	71.20
4	2415	1791	120,734,966	49.99	67.41
5	2243	1650	119,020,588	53.06	72.13
6	2437	1568	117,642,375	48.27	75.02
7	2191	1555	108,433,636	49.49	69.73
8	2351	1773	112,672,867	47.93	63.55
9	1894	1329	91,568,626	48.35	68.90
10	2098	1582	101,087,560	48.18	63.90
11	2138	1589	106,225,002	49.68	66.85
12	1749	1340	87,277,232	49.90	65.13
13	1649	1125	83,034,183	50.35	73.81
14	1911	1355	94,672,733	49.54	69.87
15	1639	1168	81,904,557	49.97	70.12
16	1592	1105	79,370,172	49.86	71.83
17	1469	1046	71,137,785	48.43	68.01
18	1291	773	67,275,902	52.11	87.03
19	1227	826	62,516,450	50.95	75.68
20	1495	1127	71,784,255	48.02	63.69
21	1430	1076	69,425,955	48.55	64.52
22	1169	820	60,283,066	51.57	73.51
23	1047	720	48,866,549	46.67	67.87
24	1323	939	62,310,066	47.10	66.35
25	855	586	42,858,509	50.13	73.13
26	1044	786	51,421,553	49.25	65.42
27	928	681	44,709,034	48.18	65.65
28	914	683	44,672,302	48.88	65.40
29	977	720	51,332,696	52.54	71.29
X	1986	559	115,943,529		
0^b^	1420	625			

^a^Derived from latest goat genome sequence assembly (ARS1) (https://www.ncbi.nlm.nih.gov/assembly/?term=Capra+hircus). ^b^Unallocated SNPs.

### Significantly associated SNPs and identified genes

**Figure 1:**
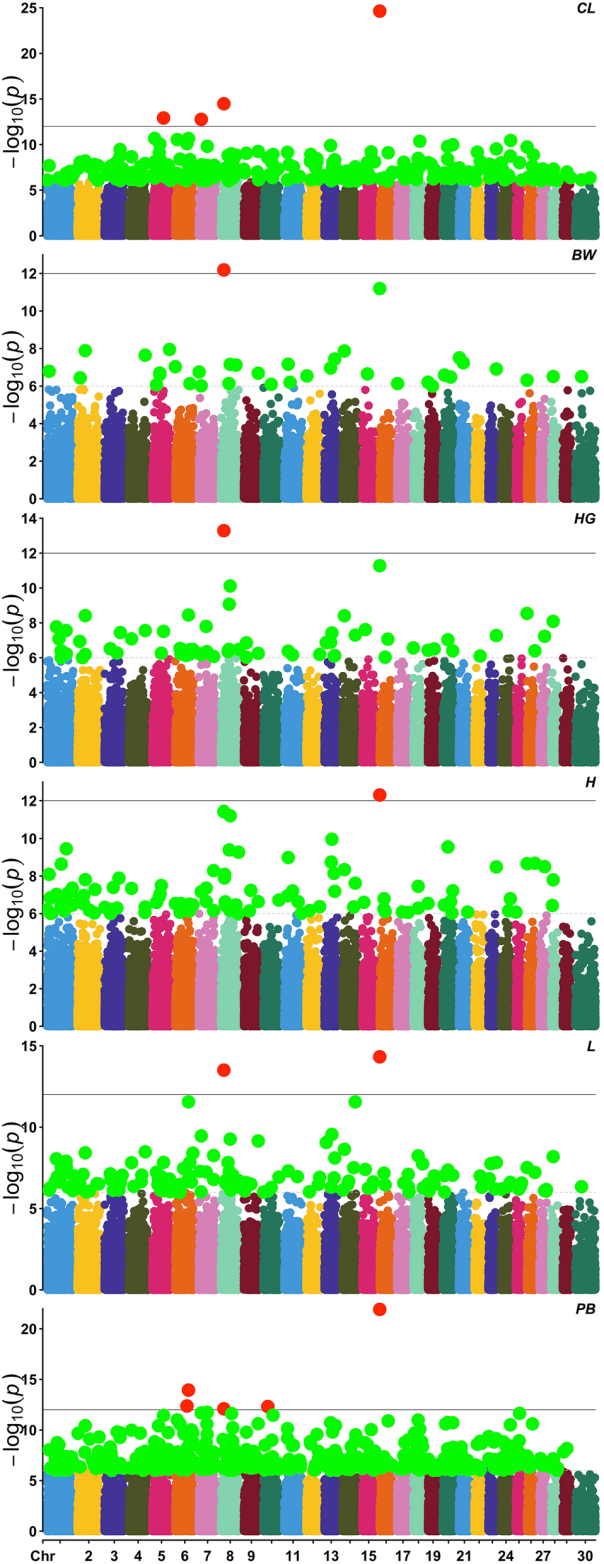
Genome wide Manhattan plot of the SNPs influencing CL = chest length, BW = body weight, HG = heart girth, H = height, L = body length, and PB = pubic bone length among Punjab goats generated using Caprine 50 K SNP Bead Chip.

**Figure 2:**
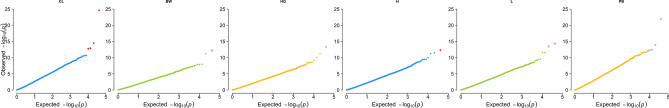
Genome wide QQ plot of the SNPs influencing CL = chest length, BW = body weight, HG = heart girth, H = height, L = body length, and PB = pubic bone length among Punjab goats using Caprine 50 K SNP Bead Chip.

Manhattan plot for body weight revealed that there were two highly significant SNPs for body weight as indicated in Table 2 and Fig. 1. The validity of *P* value was determined using quantile–quantile (QQ) plots (Fig. 2). There were two significant SNPs viz. snp24590-scaffold25-1223464 (Chromosome 8) and snp45231-scaffold617-879437 (Chromosome 16) significant deviated from the rest of the SNPs. Moreover, after False Discovery Rate with Benjamin-Hochberg procedure (FDR-BH) correction application, these SNPs remained significant for body weight as indicated in Table 2. Raw *P* values and FDR-BH corrected Stage 1 and Stage 2 *P* values of these SNPs associated with body weight and respective genes are given in Table 2. There were 914 and 798 genes in the respective regions for snp24590-scaffold25-1223464 and snp45231-scaffold617-879437 respectively (Table 2) while the closest genes in physical location were Translation initiation factor IF-2-like (IF2) and Plasma membrane calcium-transporting ATPase 4-like respectively. Manhattan and QQ plotting for body length, chest length, heart girth and pubic bone length shared these two SNPs with body weight trait (Table 2, Figs. 1, 2).

**Table 2:** Genome-wide significant SNPs with their candidate genes affecting body weight and morphometric traits among seven Punjab goat breeds.

Traits	SNPs	Chromosomes	ARS1 position (bp)	Nearest gene		Genes in the region	Raw *P* value stage 1	FDR-BH stage 1	Raw *P* value stage 2	FDR-BH stage 2	Analogous genes in mammals
				Name	Distance (bp)						
Body weight	snp24590-scaffold25-1223464	8	17901259	LOC108636659	+ 13,757	914	6.516e−13	2.737e−08	6.516e−13	5.865e−12	Translation initiation factor IF-2-like
	snp45231-scaffold617-879437	16	853228	LOC106502935	− 58,965	798	6.396e−12	1.343e−07	6.396e−12	2.878e−11	Plasma membrane calcium-transporting ATPase 4-like
Body height	snp45231-scaffold617-879437	16	853228	LOC106502935	− 58,965	798	5.013e−13	2.105e−08	6.396e−12	2.878e−11	Plasma membrane calcium-transporting ATPase 4-like
	snp24590-scaffold25-1223464	8	17901259	LOC108636659	+ 13,757	798	3.687e−12	7.743e−08	1.941e−12	1.747e−11	Translation initiation factor IF-2-like
	snp12189-scaffold1454-532653	8	55351345	TLE4	+ 24,297	914	6.176e−12	8.645e−08	5.136e−12	2.311e−11	Transducin-like enhancer protein 4
Body length	snp45231-scaffold617-879437	16	853228	LOC106502935	− 58,965	798	4.743e−15	6.545e−10	1.945e−14	1.75e−13	Plasma membrane calcium-transporting ATPase 4-like
	snp24590-scaffold25-1223464	8	17901259	LOC108636659	+ 13,757	914	3.117e-14	6.545e−10	4.216e−14	1.897e−13	Translation initiation factor IF-2-like
	snp27986-scaffold30-2051903	6	81605977	EPHA5	Within	787	2.759e−12	2.936e−08	1.501e−12	4.502e−12	EPHA5
	snp42664-scaffold566-3664570	14	15433103	LOC106502872	Within	641	2.797e−12	2.936e−08	2.043e−12	4.596e−12	Unknown gene
Chest length	snp45231-scaffold617-879437	16	853228	LOC106502935	− 58,965	798	2.312e−25	9.71e−21	2.312e−25	2.081e−24	Plasma membrane calcium-transporting ATPase 4-like
	snp24590-scaffold25-1223464	8	17901259	LOC108636659	+ 13,757	914	3.343e−15	7.019e−11	3.343e−15	1.504e−14	Translation initiation factor IF-2-like
	snp28833-scaffold310-5443065	5	67502193	ENSCHIG00000017559	Within	1498	1.22e−13	1.708e−09	1.22e−13	3.66e−13	KIAA1033
	snp2733-scaffold1079-1194419	7	15499689	RHOBTB3	Within	1557	1.726e−13	1.812e−09	1.726e−13	3.884e−13	rho-related BTB domain-containing protein 3
Heart girth	snp24590-scaffold25-1223464	8	17901259	LOC108636659	+ 13,757	914	5.144e−14	2.16e−09	5.144e−14	4.63e−13	Translation initiation factor IF-2-like
	snp45231-scaffold617-879437	16	853228	LOC106502935	− 58,965	798	5.28e−12	1.109e−07	5.28e−12	2.376e−11	Plasma membrane calcium-transporting ATPase 4-like
Pubic bone length	snp45231-scaffold617-879437	16	853228	LOC106502935	− 58,965	798	1.207e−22	5.07e−18	1.207e−22	1.087e−21	Plasma membrane calcium-transporting ATPase 4-like
	snp27986-scaffold30-2051903	6	81605977	ENSCHIG00000010390	Within	787	1.144e−14	2.403e−10	1.144e−14	5.15e−14	EPH Receptor A5 (EPHA5)
	snp58134-scaffold94-7205823	6	72533247	THEGL	− 54,447	787	4.225e−13	5.027e−09	4.225e−13	1.077e−12	Spermatid protein-like (THEGL)
	snp31031-scaffold343-1182947	10	72396016	LOC108637001	34,050	1174	4.788e−13	5.027e−09	4.788e−13	1.077e−12	Unknown ncRNA
	snp24590-scaffold25-1223464	8	17901259	LOC108636659	+ 13,757	914	7.964e−13	6.689e−09	7.964e−13	1.434e−12	Translation initiation factor IF-2-like

GWAS analysis for body height trait revealed a third significantly affecting SNP other than two aforementioned SNPs viz. snp12189-scaffold1454-532653 located on chromosome 8 mentioned in Table 2 with raw, Stage 1 and Stage 2 FDR-BH P values. There were 914 genes in the region while the closest gene was Transducin-like [transducin-like] enhancer protein 4 (TLE4) that was found + 24,297 bp away from the SNP.

Likewise, GWAS analysis for body height trait generated two additional significantly affecting SNPs other than two aforementioned SNPs on chromosome 8 and 16 viz. snp28833-scaffold310-5443065 located on chromosome 5 and snp2733-scaffold1079-1194419 located on chromosome 7 given in Table 2 with raw, Stage 1 and Stage 2 FDR-BH *P* values. There were 1498 and 1557 genes in the respective regions of these SNPs. The snp28833-scaffold310-5443065 was within WASH Complex Subunit 4 (WASHC4) gene while snp2733-scaffold1079-1194419 was located within Rho-related BTB domain-containing protein 3 (RHOBTB3) gene.

Finally, there were five SNPs identified as result of GWAS analysis for pubic bone out of which two on chromosome 8 and 16 as detailed above and mentioned in Table 2. Moreover, among three remaining SNPs one was located on chromosome 10 while two were located on chromosome 6. These SNPs were snp31031-scaffold343-1182947, snp27986-scaffold30-2051903 and snp58134-scaffold94-7205823 respectively where closest genes located were 34,050 bp away within gene and −54,447 bp away respectively. Respective genes for SNPs located on chromosome 6 were EPH Receptor A5 (EPHA5) and Spermatid protein-like (THEGL) while Unknown ncRNA for chromosome 10 SNP as detailed in Table 2 along with raw, Stage 1 and Stage 2 FDR-BH *P* values.

### Major chromosomal regions, prominent genes and polygenic risk score (PRS)

Both chromosome 6 and 8 are harboring two SNPs in the same region with 786 and 914 genes in a window of 100 MB respectively given in Table 2. Similarly, chromosome 5, 7, 10, 14 and 16 are having one SNP each with 1498, 1557, 1174, 641 and 798 genes in a window of 100 MB respectively (Table 2).

Among 786 genes in the window of 100 MB on chromosome 6; some of these play important role in growth and development i.e. Apelin Receptor Early Endogenous Ligand (APELA) is required for heart development, PR Domain Zinc Finger Protein 5 (PRDM5) is involved in cell differentiation, LIN-28 family RNA-binding protein (LIN28) is responsible for developmental timing and self-renewal in embryonic stem cells, Dickkopf WNT Signaling Pathway Inhibitor 2 (DKK2) is having a role in embryonic development, TBC1 Domain Containing Kinase (TBCK), Epidermal Growth Factor (FGF) and Ankyrin 2 (ANK2) are engaged in the regulation of cell proliferation and growth. Similarly, there were 914 genes in the window of 100 MB on chromosome 8 while genes are involved in embryogenesis i.e. GATA Binding Protein 4 (GATA4), cellular processes such as cell growth and differentiation (Protein Tyrosine Phosphatase Receptor Type D), brain development (Nuclear Factor I B), development of pancreatic beta cells, thyroid, eye, liver and kidney (GLIS Family Zinc Finger 3) and male sex determination and differentiation by controlling testis development and male germ cell proliferation (Doublesex and Mab-3 Related Transcription Factor 1).

Polygenic risk score (PRS) is given in Supplementary file that ranged between − 0.340 to 0.136, − 1.240 to 0.441, − 0.570 to 0.212, − 1.023 to 0.428, − 1.502 to 0.444, − 1.068 to 0.359 for pubic bone length, heart girth, chest length, body length, body weight and body height respectively.

## Discussion

Demand of goat meat is increasing due to ever increasing human population with improved living and national standards^16,19^. Body weight and morphometric measurements are economically important traits for genetic improvement of meat type goats^20^. Keeping in view, the rapidly increasing demand of goat meat and importance of growth trait current study was performed using Illumina 50 K SNP Bead Chip to identify SNPs and genetic variations influencing growth in term of age adjusted body weights and body conformation traits.

GWAS is widely applicable in identification of casual genes using single nucleotide polymorphism. GWAS is an ideal technique for discovery of major genes especially for complex traits and a novel way to study the genetic mechanism of these traits. Moreover, identification of genomic regions and their genes regarding economically important traits in livestock paved the way for selection of genetic markers and candidate genes which influence the trait of interest. Marker assisted selection is faster, more reliable and economical as compared to traditional selection as it enables selection of younger animals with desirable traits on the basis of highly significant genomic regions and their candidate genes. Such an approach may accelerate the breeding progress and enhance the economic prospects for selection of best breeding animals^21^.

It is quite evident from the previous studies that body weight appears to be the function of linear body measurements^22^. We identified two highly significant SNPs influencing body weight. Moreover, 2, 3, 4, 4 and 5 highly significant associations were observed for heart girth, height, body length, chest length and pubic bone length respectively. Almost more than half of these SNPs were found in the coding regions of the genes. Generally different SNPs affect different traits but this study explored two of the nine SNPs affecting more than one trait as previously stated byWu et al.^23^in GWAS of body conformation traits in Chinese Holstein cattle population. We observed that there were two SNPs (45231-scaffold617-879437 on chromosome 16 and snp24590-scaffold25-1223464 on chromosome 8) which were significantly associated with all the six traits under investigation in the current study. Moreover, candidate genes identified for growth were also influenced body conformation traits and vice versa which might be due to genetic correlation among studied traits and also because of a pleiotropic effect of these SNPs and their respective genes as previously suggested in GWAS of body conformation traits in the Chinese Holstein cattle population^23^.

In the current study, it was observed that more than half of highly significant SNPs were located in the coding regions of the genes influencing growth in term of age adjusted body weight and morphometric measurements. Moreover, current study identified a number of the most promising candidate genes which might play a critical role in growth and body conformation traits on the basis of highly significant SNPs. The genes identified in the current study may broadly be categorized as those involved in 1. cell growth and development; 2. cellular transportation, transcription and translation and 3. fertility.

The candidate genes involved in cell growth and development mechanism are EPH Receptor A5 (EPHA5), Rho-related BTB domain-containing protein 3 (RhoBTB3), Spermatid protein-like (THEGL) and Transducin-like enhancer protein 4 (TLE4). EPH Receptor A5 (EPHA5) was a candidate gene for body length in the present study. EPHA5 along with EFNA5 mediates communication between pancreatic islet cells to regulate glucose-stimulated insulin secretion. Insulin plays a key role in utilizing sugar in the body which is needed for proper growth, metabolism and tissue repair in the body secretions^24,25^. RhoBTB3 is proposed to have a role in the tissue development during embryonic life^26^while THEGL is involved in all stages of male gonads development such as sperm maturation, its fertilization and subsequent embryonic development. Moreover it has also been reported to have a high level of expression from adolescence to adulthood suggesting key role in the organs development and onset of puberty^27^. TLE4 supresses Pax7-mediated Myf5 transcriptional activation through inducing Myf5 enhancer to continue latency. Loss of TLE4 function results into Myf5 upregulation thus, showing its role in growth through transcription suppression^28^. TLE4 is also a critical regulator in haematopoiesis^29^and bone development^30^.

The candidate genes involved in cellular transportation, transcription and translation mechanism were RhoBTB3, WASH Complex Subunit 4(WASHC4), Plasma membrane calcium-transporting ATPase 4-like (ATP2B4) and Translation initiation factor IF-2-like (IF2) as appeared in current GWAS analysis.

RhoBTB3 is involved in cell cycle thus has a role in mitosis and eventually also in development as mentioned in the last paragraph^31^. WASHC4 is a gene encoding a component of WASH complex that is responsible for transport of endosomes within the cell^32^; moreover, a mutation of this gene resulted in remarkable developmental disorders and skeletal muscles dysmorphism^33^. ATP2B4 is an enzyme that is calcium/calmodulin-regulated and magnesium-dependent. It catalyzes the hydrolysis of ATP along with the calcium transportation out of cell as well as has a role in sperm motility^34^. Translation initiation factor 2 (IF2) is required for GTP/GDP-binding protein whose principal role is to interact with initiator fMet-tRNA and to position it correctly in the ribosomal P site, thus enhancing the rate and fidelity of translation initiation^35^.

The candidate genes involved in fertility mechanism are Spermatid protein-like (THEGL), Plasma membrane calcium-transporting ATPase 4-like whose role is discussed previously.

The functional aspect of aforementioned genes have been predominantly studied in human beings. However, it is suggested that these genes or their variants most probably have had more or less similar functions in other mammals i.e., goats. Moreover, in the current GWAS analysis number of highly significant SNPs were identified representing eight the most promising candidate genes which might play a key role in the growth in term of age adjusted body weight and morphometric measurements in goat. Moreover, these candidate genes seem to play a key role in growth, metabolism, cellular transportation, and fertility. This study also suggested pleiotropic effects for body weight and conformation traits of couple of SNPs and their candidate genes.

## Methods

### Ethics declarations

This study involved a questionnaire-based survey of farmers as well as blood sampling and phenotypic data recording from their animals. Participants provided their verbal informed consent for animal blood sampling as well as for the related survey questions. Collection of blood samples and all methods were performed in accordance with the relevant guidelines and regulations for animals by veterinarians adhering to the regulations and guidelines on animal husbandry and welfare as per international norms as per approved experimental protocols by the institutional Ethics Committee of PMAS-Arid Agriculture University, Rawalpindi, Pakistan. Moreover questioning of farmers were performed in accordance with the relevant guidelines and regulations for human subjects as per approved experimental protocols by institutional Ethics Committee of PMAS-Arid Agriculture University, Rawalpindi, Pakistan.

### Sampling

**Figure 3:**
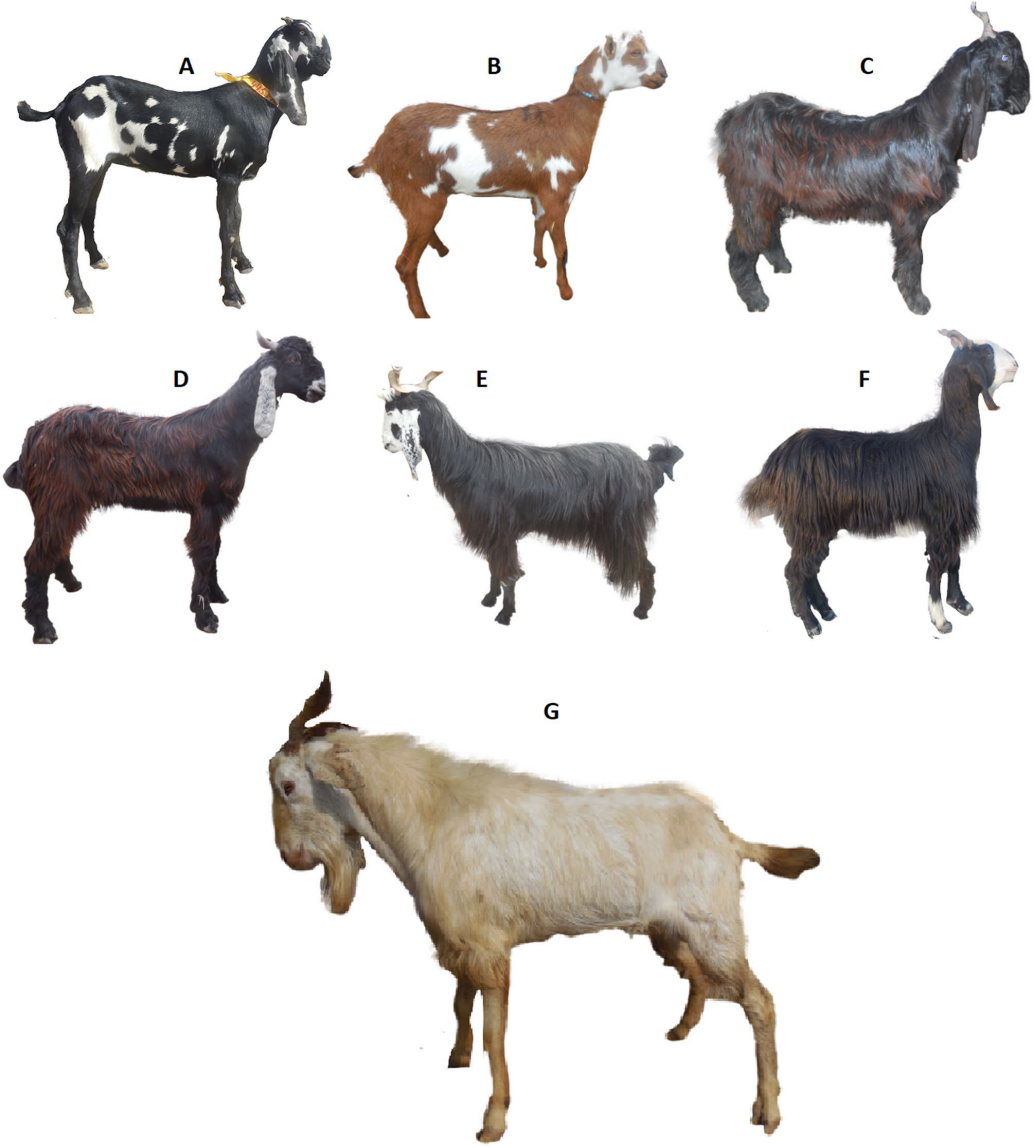
Documented goat breeds of Punjab Pakistan A = Beetal, B = Barbari, C = Daira Deen Panah, D = Nachi, E = Pahari, F = Local Pothwari and G = Teddi.

**Figure 4:**
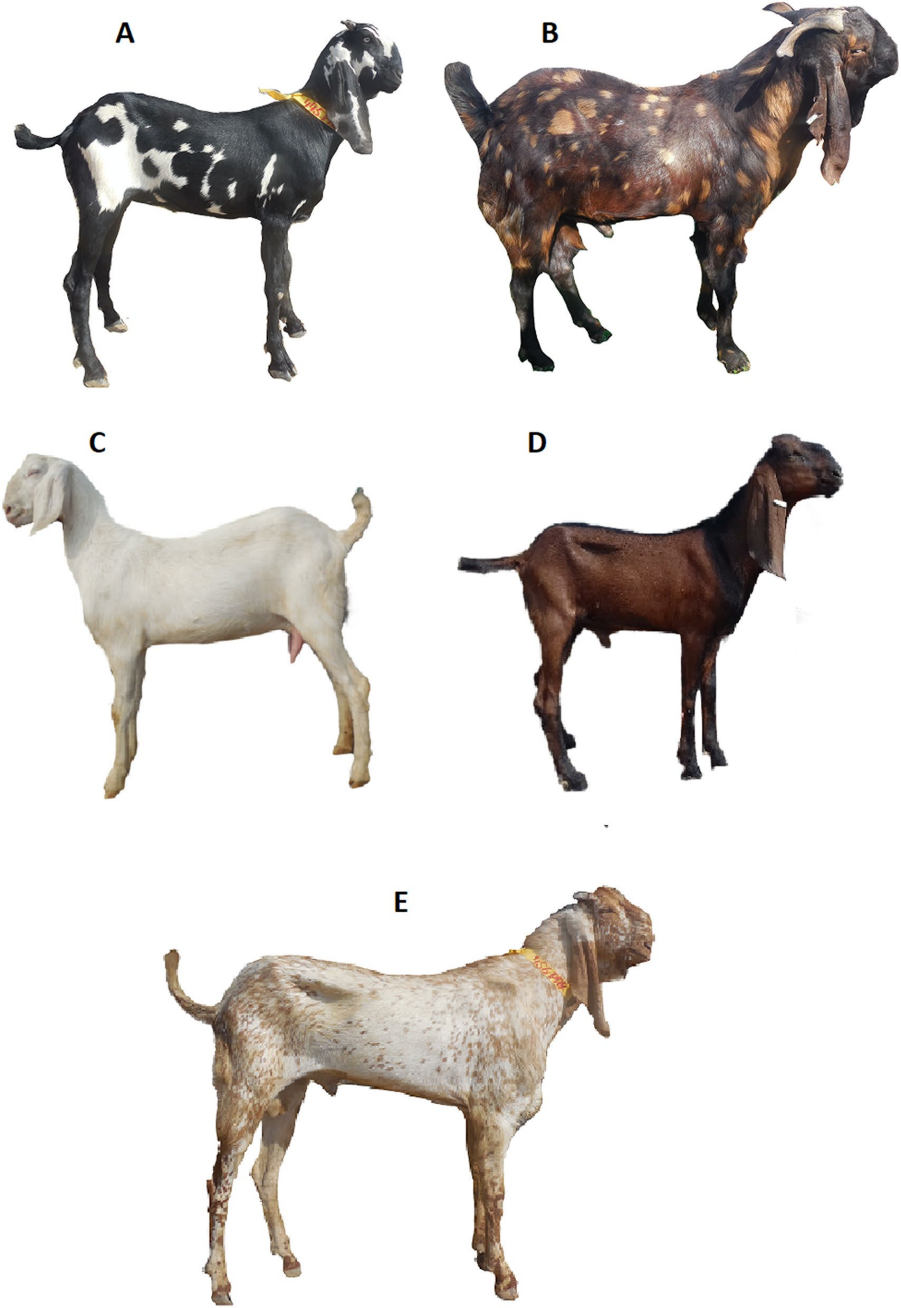
Five strains of Beetal breed A = Faislabadi, B = Gujrati, C = Nukri, D = Nagri and E = MakhiChini.

Blood samples were collected by jugular vein puncture in EDTA (Ethylene Diamine Tetra Acetate) containing vacutainers. Total of 879 samples were obtained from all the Punjab goat breeds of Pakistan namely Beetal breed including all five strain under study (Beetal Faislabadi, Beetal Nuqri, Beetal Nagri, Beetal Gujrati and Beetal MakhiChini (*N* = 631), Teddi (*N* = 114), Daira Din Panah (*N* = 21), Nachi (*N* = 33), Barbari (*N* = 23), Pahari/Kajli (*N* = 41) and Pothwari (*N* = 16). Data were collected from various districts of Punjab i.e., Fateh Jhang, Bakhar, Liyyah, D.G. Khan, Rajanpur, Faisalabad and Jhang as mentioned in Table 3. Each goat was assigned a particular ID for blood collection, body weight and linear body measurements. Sampling was done by ensuring that the sampled individuals were unrelated and there was effective representation of each breed^36^. The pictures of seven documented goat breeds of Punjab and five strains of Beetal are given in Figs. 3 and 4. Finally study was carried out in compliance with the ARRIVE guidelines.

**Table 3:** Goat breeds of Punjab with their home tract.

S. no.	Breed name	Home tract	Sampling area	Purpose	Salient breed characteristics
1	Beetal (5 strains)	Gujrat and Rawalpindi, Bahawalnagar, Faisalabad, Sheikhupura and Lahore, D.G. Khan	Faisalabad, Khanewal, DG Khan, Rajanpur, Bahawalnagar and government farms in area	Meat and Milk	Large size breed with massive head, roman nose, long legs and ears. There exist 6 strains of Beetal breed
2	Daira Din Panah	Layyah, Muzaffargarh to Multan	Muzaffargarh and Layyah, government farms in area	Meat and Milk	Black hairy goats with broad, long and spiral horns, roman nose and rough hairs on body
3	Pahari (Kajli)	D.G. Khan	D.G. Khan and government farms in area	Meat	Hairy and muscular body with small head and face, medium to large ears and spiral horns
4	Nachi (Bikaneri)	Muzaffargarh, Layyah to Multan	Multan and government farms in area	Meat	Dancing gate, long and droopy ears with predominantly black coat color
5	Barbari	D.G. Khan, Rajanpur	D.G. Khan, Rajanpur and government farms in area	Meat and Milk	Small head with straight and erect ears, mostly polled with developed udder and teats, resembles a Deer
6	Teddy	Central Punjab	Home tract and government farms in Layyah	Meat and Milk	Medium size body with small droopy ears and short conical teats
7	Pothwari	Pothohar area (Jhelum, Chakwal, Rawalpindi and Attock)	Chakwal, Attock and government farm in area	Meat and Milk	Hairy body with small legs having wide, spiral and thin horns

### Linear body measurements and body weight

Linear body measurements from all the breeds were taken which include body length, body height, pubic bone length, heart girth and chest length in centimeter (cm) as described previously^16,37^. Body weight was taken in Kilogram using weighing scale from all animals sampled.

### DNA extraction

DNA was extracted using organic method of DNA extraction as previously reported^38^. Briefly, 1 mL of blood was taken and 2 mL of lysis buffer was added. It was then vortexed and mixed well and samples were centrifuged for 5 min at 1500 g (2690 RPM). Previous step was repeated with 3 mL of Lysis buffer. Supernatant was discarded and 300 uL of DNA extraction buffer was added followed by addition of 5 µL of 10 mg/mL Proteinase K and 20 uL of 10% SDS**.** Samples were vortexed gently and kept at 65 ℃ in the incubator overnight. After that, samples were placed in − 20 ℃ freezer until completely frozen. Samples were thawed after freezing and 120 uL of 5 M NaCl was added and mixed. Samples were centrifuged for 15 min at the rate of 6000 RPM and 1 mL of 100% Ethanol was added in new conical tube while clear solution was added to get DNA in 50 uL of DNase/RNase free water. Biological samples of DNA were kept at 4 ℃ to resuspend overnight and then frozen at − 80 ℃. Concentration and optical density of DNA samples were measured using Nanodrop (Quawell Q 5000).

### Genotyping and quality control measures

All 879 samples of DNA having optimal concentration and density were then genotyped by GeneSeek USA using illumina 50 K SNP Bead Chip which is a medium density chip as it covers 53,347 SNPs spread across the whole Caprine genome^5^.

Quality control measures were performed in Plink by setting call rate of 0.95 and SNPs with minor allele frequencies (MAF) lower than 0.05 or that do not conform to the Hardy–Weinberg expectation (*P*value ≤ 0.001)^39^. This retained 827 individuals out of total 879 after applying quality control filters.

### Two stage association for body weight and morphometric measurements and polygenic risk score (PRS) estimation

After pruning and quality control measures two stage SNPs association analysis was carried out as methodology adapted from Shi et al.^40^. In Stage 1, association study was performed for age adjusted body weights in Punjab goat breeds. Same procedure was used for identifying SNPs and respective genes or genomic regions regarding morphometric measurements. Linear model was used for finding significant SNPs influencing body weight. Based on False Discovery Rate of Benjamini and Hochberg test (FDR-BH); a significance level was defined as 10e−8 for the traits under study. The identified SNPs were further carried to Stage 2 of analysis on all animals. Identified SNPs were used to calculate polygenic risk score (PRS) using Genome-wide Complex Trait Analysis (GCTA)^41^and Plink 1.9^42^.

### Manhattan and quantile quantile plots

Manhattan and Quantile Quantile **(**QQ) plots were made in R program using CMplot package (https://github.com/YinLiLin/CMplot). For SNPs with *P* values less than 10e−8, the Q–Q plot represented highly significant deviations from the distribution under the null hypothesis indicating a strong association of these SNPs and respective regions with body weight and morphometric measurements.

### Genes annotation

Genes annotation was performed with latest goat genome assembly *Capra hircus* ARS1. We identified genes listed on www.genecards.org that were functionally responsible for the phenotypic effects.

### Statistical analysis

Morphometric measurements data i.e. body weight, body length, body height, pubic bone length, heart girth and chest length were analyzed using Proc mixed of SAS University Edition. Any wrong and biologicaly impossible information was not included in the final analysis. Raw data mean, standard deviation, minimum and maximum values for the studied traits are given in Table 4.

**Table 4:** Raw Mean, standard deviation, minimum and maximum values for age (months), body weight (kg), heart girth (cm), height (cm), length (cm), pubic bone length (cm) and chest length (cm).

Variable	No of samples	Mean	SD	Minimum	Maximum
Age (days)	827	719.25	442.84	Birth age	2580 days
Body weight (kg)	827	39.77	16.19	1.5	115.00
Heart girth (cm)	827	76.33	12.12	24.00	110.00
Wither height (cm)	827	76.88	11.52	12.00	111.00
Body length (cm)	827	67.73	10.51	19.00	96.00
Pubic bone length (cm)	827	11.93	2.69	3.00	21.50
Chest length (cm)	827	20.47	4.40	6.00	39.00

The mixed linear model was used to describe the data as previously reported^43^ and model equation narrated as under.

$$y_{i} = \gamma age + X\alpha + Z\beta + W\mu + \varepsilon_{i}$$

where $y_{i}$ is the phenotypic value of the *i*th individual, $age$ is the effect of age in days on body weight while $\gamma$ is regression coefficient, $\alpha$ is the fix effect of breed including Beetal, Teddi, Daira Din Panah, Pahari, Pothwari and Barbari and sex of each individual including male and female. Z indicates matrix of SNP effect, $\beta$ is SNP effect vector, W is indicator of polygenic residual, µ is the polygenic residual effect vector and ${\upvarepsilon }_{{\text{i}}}$ is the residual with variance $\sigma_{\varepsilon }^{2}$.

## Supplementary Information

Supplementary Information.

## Data Availability

The datasets generated and/or analysed during the current study are available in the figshare.com repository and can be accessed at https://figshare.com/articles/dataset/Genome_wide_association_study_identifies_novel_candidate_genes_for_growth_and_body_conformation_traits_in_goats/19668633.
