# Author Correction: Genome wide association study identifies novel candidate genes for growth and body conformation traits in goats

**DOI:** 10.1038/s41598-025-99916-7

**Published:** 2025-05-13

**Authors:** Muhammad Moaeen-ud-Din, Raja Danish Muner, Muhammad Sajjad Khan

**Affiliations:** 1 Department of Animal Breeding and Genetics, Faculty of Veterinary and Animal Sciences, PMAS-Arid Agriculture University, Rawalpindi, 46300 Pakistan; 2 Cholistan University of Veterinary and Animal Sciences, Bahawalpur, 63100 Pakistan

Correction to: *Scientific Reports* 10.1038/s41598-022-14018-y, published online 14 June 2022

The original version of this Article contained errors.

Reference 24 was incorrect and has now been removed. The subsequent References have been re-numbered.

As a result, in the Discussion section,

“EPHA5 along with EFNA5 mediates communication between pancreatic islet cells to regulate glucose-stimulated insulin secretion. Insulin plays a key role in utilizing sugar in the body which is needed for proper growth, metabolism and tissue repair in the body secretions^24,25,26^.”

now reads:

“EPHA5 along with EFNA5 mediates communication between pancreatic islet cells to regulate glucose-stimulated insulin secretion. Insulin plays a key role in utilizing sugar in the body which is needed for proper growth, metabolism and tissue repair in the body secretions^24,25^.”

Original References

24. Vickers, N. J. Animal communication: When i’m calling you, will you answer too?. Curr. Biol. 27, R713–R715 (2017).

25. Jain, R. et al. Pharmacological inhibition of Eph receptors enhances glucose-stimulated insulin secretion from mouse and human pancreatic islets. Diabetologia 56, 1350–1355 (2013).

26. Konstantinova, I. et al. EphA-Ephrin-A-mediated β cell communication regulates insulin secretion from pancreatic islets. Cell 129, 359–370 (2007).

The original Article has been corrected.

